# (6*Z*)-4-Bromo-6-{[(2-hy­droxy­eth­yl)amino]­methyl­idene}cyclo­hexa-2,4-dien-1-one

**DOI:** 10.1107/S1600536812009749

**Published:** 2012-03-10

**Authors:** Shaaban K. Mohamed, Mehmet Akkurt, Antar A. Abd Elhamid, Kuldip Singh, Herman Potgieter

**Affiliations:** a Chemistry and Environmental Science Division, School of Science, Manchester Metropolitan University, England; b Department of Physics, Faculty of Sciences, Erciyes University, 38039 Kayseri, Turkey; c Department of Chemistry, University of Leicester, Leicester, England; d School of Research, Enterprise & Innovation, Manchester Metropolitan University, England

## Abstract

The title mol­ecule, C_9_H_10_BrNO_2_, excluding methyl­ene H atoms and the C—OH group, is essentially planar, with a maximum deviation of 0.037 (2) Å for the N atom. The N—C—C—O torsion angle is −63.1 (3)°. The mol­ecular structure is stabilized by a weak intra­molecular N—H⋯O(carbonyl) hydrogen bond, forming an *S*(6) motif. In the crystal, mol­ecules are linked by O—H⋯O and C—H⋯O hydrogen bonds, forming a three-dimensional network.

## Related literature

For background to amino­alcohol derivatives and their bioactivity, see: Thomas *et al.* (1990 ▶); Rubinstein & Svendsen (1994 ▶); Erdemir (2012 ▶). For the synthesis of a similar structure, see: Chakravarthy & Chand (2011 ▶). For reference bond-length data, see: Allen *et al.* (1987 ▶). For hydrogen-bond motifs, see: Bernstein *et al.* (1995 ▶); Etter *et al.* (1990 ▶).
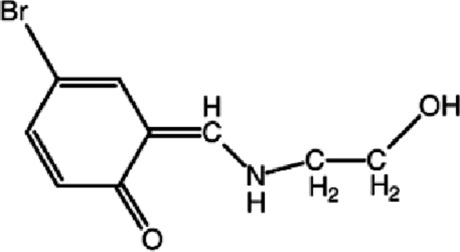


## Experimental

### 

#### Crystal data

C_9_H_10_BrNO_2_
*M* *_r_* = 244.08
Monoclinic, 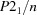

*a* = 4.4534 (17) Å
*b* = 11.523 (4) Å
*c* = 18.212 (7) Å
β = 95.703 (7)°
*V* = 930.0 (6) Å^3^
*Z* = 4
Mo *K*α radiation
μ = 4.39 mm^−1^
*T* = 150 K
0.25 × 0.15 × 0.05 mm

#### Data collection

Bruker APEX 2000 CCD area-detector diffractometer
Absorption correction: multi-scan (*SADABS*; Sheldrick, 1996 ▶) *T*_min_ = 0.407, *T*_max_ = 0.811
7386 measured reflections
1930 independent reflections
1442 reflections with *I* > 2σ(*I*)
*R*_int_ = 0.078

#### Refinement

*R*[*F* ^2^ > 2σ(*F* ^2^)] = 0.041
*wR*(*F* ^2^) = 0.092
*S* = 0.96
1930 reflections
119 parameters
H-atom parameters constrained
Δρ_max_ = 0.50 e Å^−3^
Δρ_min_ = −0.81 e Å^−3^

### 

Data collection: *APEX2* (Bruker, 2005 ▶); cell refinement: *SAINT* (Bruker, 2005 ▶); data reduction: *SAINT*; program(s) used to solve structure: *SHELXS97* (Sheldrick, 2008 ▶); program(s) used to refine structure: *SHELXL97* (Sheldrick, 2008 ▶); molecular graphics: *ORTEP-3 for Windows* (Farrugia, 1997 ▶) and *PLATON* (Spek, 2009 ▶); software used to prepare material for publication: *WinGX* (Farrugia, 1999 ▶) and *PLATON*.

## Supplementary Material

Crystal structure: contains datablock(s) global, I. DOI: 10.1107/S1600536812009749/wn2469sup1.cif

Structure factors: contains datablock(s) I. DOI: 10.1107/S1600536812009749/wn2469Isup2.hkl

Supplementary material file. DOI: 10.1107/S1600536812009749/wn2469Isup3.cml

Additional supplementary materials:  crystallographic information; 3D view; checkCIF report

## Figures and Tables

**Table 1:** Hydrogen-bond geometry (Å, °)

*D*—H⋯*A*	*D*—H	H⋯*A*	*D*⋯*A*	*D*—H⋯*A*
N1—H1⋯O1	0.86	1.91	2.581 (3)	134
O2—H2*A*⋯O1^i^	0.82	1.86	2.672 (4)	173
C9—H9*B*⋯O1^ii^	0.97	2.54	3.341 (4)	140

Symmetry codes: (i) 
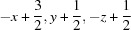
; (ii) 
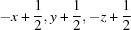
.

**Fig. 1.**
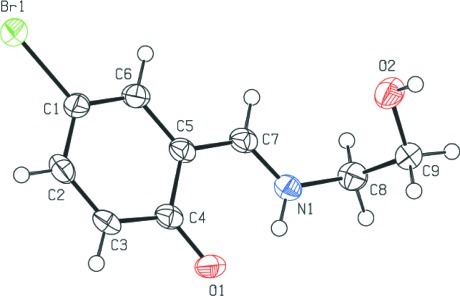
The molecular structure, showing displacement ellipsoids drawn at the 50% probability level. Hydrogen atoms are shown as spheres of arbitrary radius.

**Fig. 2.**
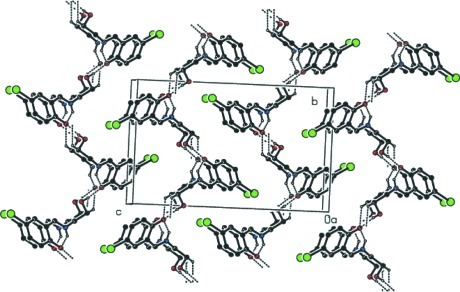
View of the packing down the a axis. Dashed lines indicate hydrogen bonds. H atoms not involved in hydrogen bonding have been omitted for clarity.

## supplementary crystallographic information

### Comment

The amino alcohol functionality is present in many classes of compounds having chemotherapeutic activity (Erdemir, 2012; Rubinstein & Svendsen, 1994; Thomas *et al.*, 1990). In addition, phenolic compounds containing the aminoalcohol grouping in *ortho* positions act as excellent bidentate ligands for the formation of several metal complexes (Chakravarthy & Chand, 2011).

As an extension of our work on the reactivity of primary aminoalcohols in three-component reactions, the title compound has been isolated as a secondary product from the one-pot reaction of (2*E*)-3-(4-methylphenyl)-1-phenylprop-2-en-1-one (chalcone), 5-bromo-2-hydroxybenzaldehyde and aminoethanol under mild conditions.

As shown in Fig. 1, excluding methylene H atoms and the C—OH group, the molecule is essentially planar, with a maximum deviation of 0.037 (2) Å for N1. The N1—C8—C9—O2 torsion angle is -63.1 (3)°. The bond lengths (Allen *et al.*, 1987) and angles have normal values.

The molecular structure is stabilized by a weak intramolecular N—H···O hydrogen bond, which generates an S(6) ring motif (Bernstein *et al.*, 1995; Etter *et al.*, 1990). In addition, intermolecular O—H···O and C—H···O hydrogen bonds (Table 1, Fig. 2) contribute to the stability of the crystal structure, linking the molecules into a three-dimensional network.

### Experimental

The title compound has been obtained as a secondary product from a multicomponent reaction mixture of (2E)-3-(4-methylphenyl)-1-phenylprop-2-en-1-one (0.01mol), 5-bromo-2-hydroxybezaldehyde (0.01mol) and aminoethanol (0.01mol). The mixture was heated at 353 K in ethanol for 4 h, monitored by TLC until the reaction was completed and then cooled to room temperature. The solvent was evaporated under vacuum and the residual oil was triturated with water to afford a brown precipitate which was filtered off, washed with water and dried in a desiccator. Pale yellow plate crystals for x-ray diffraction were obtained by dissolving the product in ethanol at room temperature and leaving it to evaporate slowly over four days. 43% yield; m.p. 355 K.

### Refinement

H atoms were positioned geometrically and refined using as riding model with Csp^2^—H = 0.93 Å, C(methylene)—H = 0.97 Å, O—H = 0.82 Å and N—H = 0.86 Å; *U*_iso_(H) = x*U*_eq_(C,N,O), where x = 1.5 for hydroxyl H and 1.2 for all other H atoms.

### Crystal data

**Table:**

C_9_H_10_BrNO_2_	*F*(000) = 488
*M**_r_* = 244.08	*D*_x_ = 1.743 Mg m^−^^3^
Monoclinic, *P*2_1_/*n*	Mo *K*α radiation, λ = 0.71073 Å
Hall symbol: -P 2yn	Cell parameters from 972 reflections
*a* = 4.4534 (17) Å	θ = 3.5–28.3°
*b* = 11.523 (4) Å	µ = 4.39 mm^−^^1^
*c* = 18.212 (7) Å	*T* = 150 K
β = 95.703 (7)°	Plate, pale yellow
*V* = 930.0 (6) Å^3^	0.25 × 0.15 × 0.05 mm
*Z* = 4	

### Data collection

**Table:**

Bruker APEX 2000 CCD area-detector diffractometer	1930 independent reflections
Radiation source: fine-focus sealed tube	1442 reflections with *I* > 2σ(*I*)
Graphite monochromator	*R*_int_ = 0.078
phi and ω scans	θ_max_ = 26.5°, θ_min_ = 2.1°
Absorption correction: multi-scan (*SADABS*; Sheldrick, 1996)	*h* = −5→5
*T*_min_ = 0.407, *T*_max_ = 0.811	*k* = −14→14
7386 measured reflections	*l* = −22→22

### Refinement

**Table:**

Refinement on *F*^2^	Primary atom site location: structure-invariant direct methods
Least-squares matrix: full	Secondary atom site location: difference Fourier map
*R*[*F*^2^ > 2σ(*F*^2^)] = 0.041	Hydrogen site location: inferred from neighbouring sites
*wR*(*F*^2^) = 0.092	H-atom parameters constrained
*S* = 0.96	*w* = 1/[σ^2^(*F*_o_^2^) + (0.0441*P*)^2^] where *P* = (*F*_o_^2^ + 2*F*_c_^2^)/3
1930 reflections	(Δ/σ)_max_ = 0.001
119 parameters	Δρ_max_ = 0.50 e Å^−^^3^
0 restraints	Δρ_min_ = −0.81 e Å^−^^3^

### Special details

**Table:**

Geometry. Bond distances, angles *etc*. have been calculated using the rounded fractional coordinates. All su's are estimated from the variances of the (full) variance-covariance matrix. The cell e.s.d.'s are taken into account in the estimation of distances, angles and torsion angles
Refinement. Refinement on *F*^2^ for ALL reflections except those flagged by the user for potential systematic errors. Weighted *R*-factors *wR* and all goodnesses of fit *S* are based on *F*^2^, conventional *R*-factors *R* are based on *F*, with *F* set to zero for negative *F*^2^. The observed criterion of *F*^2^ > σ(*F*^2^) is used only for calculating -*R*-factor-obs *etc*. and is not relevant to the choice of reflections for refinement. *R*-factors based on *F*^2^ are statistically about twice as large as those based on *F*, and *R*-factors based on ALL data will be even larger.

### Fractional atomic coordinates and isotropic or equivalent isotropic displacement parameters (Å^2^)

**Table:**

	*x*	*y*	*z*	*U*_iso_*/*U*_eq_	
Br1	1.18634 (9)	0.87511 (3)	0.62311 (2)	0.0421 (1)	
O1	0.7253 (5)	0.62015 (18)	0.34691 (14)	0.0337 (7)	
O2	0.4433 (6)	1.0017 (2)	0.24200 (13)	0.0405 (8)	
N1	0.3527 (5)	0.7886 (2)	0.31526 (14)	0.0281 (8)	
C1	1.0339 (8)	0.7961 (3)	0.53566 (17)	0.0307 (10)	
C2	1.1550 (7)	0.6880 (3)	0.51979 (19)	0.0328 (11)	
C3	1.0572 (8)	0.6302 (3)	0.45664 (19)	0.0306 (10)	
C4	0.8236 (7)	0.6747 (3)	0.40514 (19)	0.0267 (9)	
C5	0.7034 (7)	0.7865 (3)	0.42349 (17)	0.0263 (10)	
C6	0.8125 (7)	0.8450 (3)	0.48844 (18)	0.0289 (10)	
C7	0.4695 (7)	0.8372 (3)	0.37491 (18)	0.0277 (10)	
C8	0.1263 (7)	0.8437 (3)	0.26310 (19)	0.0326 (11)	
C9	0.2721 (8)	0.9117 (3)	0.20525 (18)	0.0314 (11)	
H1	0.41130	0.71970	0.30550	0.0340*	
H2	1.30420	0.65520	0.55270	0.0390*	
H2A	0.54910	1.03260	0.21320	0.0610*	
H3	1.14560	0.55950	0.44680	0.0370*	
H6	0.73370	0.91700	0.49920	0.0350*	
H7	0.39700	0.90970	0.38700	0.0330*	
H8A	0.00310	0.89540	0.28960	0.0390*	
H8B	−0.00450	0.78470	0.23920	0.0390*	
H9A	0.40180	0.86150	0.17950	0.0380*	
H9B	0.11890	0.94400	0.16950	0.0380*	

### Atomic displacement parameters (Å^2^)

**Table:**

	*U*^11^	*U*^22^	*U*^33^	*U*^12^	*U*^13^	*U*^23^
Br1	0.0616 (3)	0.0314 (2)	0.0309 (2)	−0.0066 (2)	−0.0074 (2)	0.0005 (2)
O1	0.0400 (13)	0.0243 (12)	0.0361 (13)	0.0005 (10)	0.0008 (10)	−0.0076 (11)
O2	0.0487 (15)	0.0422 (15)	0.0306 (13)	−0.0192 (12)	0.0033 (11)	−0.0005 (12)
N1	0.0275 (15)	0.0242 (14)	0.0326 (15)	−0.0018 (11)	0.0035 (12)	0.0017 (12)
C1	0.0412 (19)	0.0245 (17)	0.0261 (17)	−0.0088 (15)	0.0017 (14)	0.0002 (14)
C2	0.0353 (19)	0.0257 (18)	0.0366 (19)	−0.0015 (15)	−0.0010 (15)	0.0105 (15)
C3	0.0368 (18)	0.0183 (15)	0.0369 (19)	−0.0004 (15)	0.0045 (14)	0.0048 (14)
C4	0.0269 (16)	0.0221 (16)	0.0321 (17)	−0.0048 (14)	0.0080 (13)	0.0021 (15)
C5	0.0285 (17)	0.0216 (16)	0.0295 (17)	−0.0022 (13)	0.0067 (13)	0.0029 (13)
C6	0.0352 (18)	0.0221 (17)	0.0300 (17)	−0.0014 (14)	0.0068 (14)	−0.0014 (13)
C7	0.0304 (17)	0.0227 (16)	0.0309 (17)	−0.0036 (14)	0.0080 (14)	0.0012 (14)
C8	0.0264 (17)	0.0322 (19)	0.0382 (19)	−0.0009 (14)	−0.0023 (14)	−0.0010 (16)
C9	0.0357 (19)	0.0294 (18)	0.0286 (18)	−0.0004 (15)	0.0008 (14)	−0.0013 (14)

### Geometric parameters (Å, º)

**Table:**

Br1—C1	1.900 (3)	C5—C6	1.406 (5)
O1—C4	1.272 (4)	C5—C7	1.423 (5)
O2—C9	1.415 (4)	C8—C9	1.511 (5)
O2—H2A	0.8200	C2—H2	0.9300
N1—C8	1.460 (4)	C3—H3	0.9300
N1—C7	1.285 (4)	C6—H6	0.9300
N1—H1	0.8600	C7—H7	0.9300
C1—C2	1.399 (5)	C8—H8A	0.9700
C1—C6	1.364 (5)	C8—H8B	0.9700
C2—C3	1.363 (5)	C9—H9A	0.9700
C3—C4	1.425 (5)	C9—H9B	0.9700
C4—C5	1.447 (5)		
			
C9—O2—H2A	109.00	C1—C2—H2	120.00
C7—N1—C8	123.8 (3)	C3—C2—H2	120.00
C7—N1—H1	118.00	C2—C3—H3	119.00
C8—N1—H1	118.00	C4—C3—H3	119.00
C2—C1—C6	120.5 (3)	C1—C6—H6	120.00
Br1—C1—C2	119.1 (2)	C5—C6—H6	120.00
Br1—C1—C6	120.4 (3)	N1—C7—H7	118.00
C1—C2—C3	120.8 (3)	C5—C7—H7	118.00
C2—C3—C4	122.1 (3)	N1—C8—H8A	109.00
O1—C4—C3	122.6 (3)	N1—C8—H8B	109.00
O1—C4—C5	121.9 (3)	C9—C8—H8A	109.00
C3—C4—C5	115.5 (3)	C9—C8—H8B	109.00
C6—C5—C7	119.8 (3)	H8A—C8—H8B	108.00
C4—C5—C6	121.1 (3)	O2—C9—H9A	110.00
C4—C5—C7	119.1 (3)	O2—C9—H9B	110.00
C1—C6—C5	120.0 (3)	C8—C9—H9A	110.00
N1—C7—C5	123.8 (3)	C8—C9—H9B	110.00
N1—C8—C9	111.3 (3)	H9A—C9—H9B	108.00
O2—C9—C8	107.4 (3)		
			
C8—N1—C7—C5	−176.0 (3)	O1—C4—C5—C6	179.3 (3)
C7—N1—C8—C9	89.7 (4)	C3—C4—C5—C7	180.0 (3)
Br1—C1—C6—C5	179.3 (2)	O1—C4—C5—C7	−0.3 (5)
C2—C1—C6—C5	0.3 (5)	C3—C4—C5—C6	−0.5 (5)
Br1—C1—C2—C3	−178.2 (3)	C4—C5—C6—C1	−0.5 (5)
C6—C1—C2—C3	0.8 (5)	C6—C5—C7—N1	−178.5 (3)
C1—C2—C3—C4	−1.8 (5)	C7—C5—C6—C1	179.2 (3)
C2—C3—C4—O1	−178.2 (3)	C4—C5—C7—N1	1.1 (5)
C2—C3—C4—C5	1.6 (5)	N1—C8—C9—O2	−63.1 (3)

### Hydrogen-bond geometry (Å, º)

**Table:**

*D*—H···*A*	*D*—H	H···*A*	*D*···*A*	*D*—H···*A*
N1—H1···O1	0.86	1.91	2.581 (3)	134
O2—H2*A*···O1^i^	0.82	1.86	2.672 (4)	173
C9—H9*B*···O1^ii^	0.97	2.54	3.341 (4)	140

Symmetry codes: (i) −*x*+3/2, *y*+1/2, −*z*+1/2; (ii) −*x*+1/2, *y*+1/2, −*z*+1/2.
